# A Case of Low Rectovaginal Fistula of Obstetric Origin: Treatment by Fistulotomy and Reconstitution or Advancement Flap?

**DOI:** 10.3389/fsurg.2020.00002

**Published:** 2020-02-18

**Authors:** Elroy Patrick Weledji, Felix Adolphe Elong, Divine Enoru Eyongeta

**Affiliations:** ^1^Department of Surgery, Faculty of Health Sciences, University of Buea, Buea, Cameroon; ^2^Department of Obstetrics and Gynaecology, Faculty of Health Sciences, University of Buea, Buea, Cameroon; ^3^Department of Surgery, Faculty of Health Sciences, University of Buea, Buea, Cameroon

**Keywords:** fistula, rectovaginal, fistulotomy, composite repair, advancement flap

## Abstract

Many small low rectovaginal fistulas represent incompletely healed (third degree) perineal lacerations i. e., involving the sphincters. An individualized, systematic approach to these fistulas based on their size, location, and etiology provides a more concise treatment plan. We report a case of a low rectovaginal fistula developed some years following forceps vaginal delivery. This was managed successfully by a fistulotomy in which the bridge of skin and scar tissue was divided, and the defect repaired as a classical third degree perineal laceration. On the background of coexisting or occult sphincter damage which usually follows obstetric trauma, a fistulotomy and immediate composite repair for small, low rectovaginal fistula may be advantageous and acceptable in a low resource setting where endoanal imaging and manometry are not available.

## Background

Simple rectovaginal fistulas consist of small, low fistulas secondary to infection or trauma, but complex if large (>2.5 cm), high or caused by inflammatory bowel disease (1). They can be bothersome to both the patient (passage of flatus and liquid stool per vaginum), and the surgeon (high failure rate after repair) (1, 2). Many of these patients have a coexistent or occult sphincter injury, and the symptoms of rectovaginal fistula may mask fecal incontinence that may manifest when the fistula is repaired (2). In low fistulas, the rectal defect is in the dentate line with the vaginal opening inside the vaginal fourchette. Simple rectovaginal fistulas generally have healthy, vascularized surrounding tissue, which can be repaired with local techniques including a fistulotomy and immediate reconstitution or an advancement flap. A perineal, transanal, or transvaginal approach is usually suitable for the majority of low fistulas (1, 3). The work has been reported in line with the SCARE 2018 criteria (4).

## Case Presentation

A patient in the age range of 30–35 years was referred with a 1 year history of fecal drainage through the vagina following the passage of soft stool but no frank incontinence. She had an unrecognized injury from forceps vaginal delivery and a repaired perineal tear 5 years previously. She had no history of constipation that could cause a stercoral rectovaginal fistula, anorectal sepsis, inflammatory bowel disease, anorectal, or pelvic surgery. On examination, she was clinically well.

**Figure 1:**
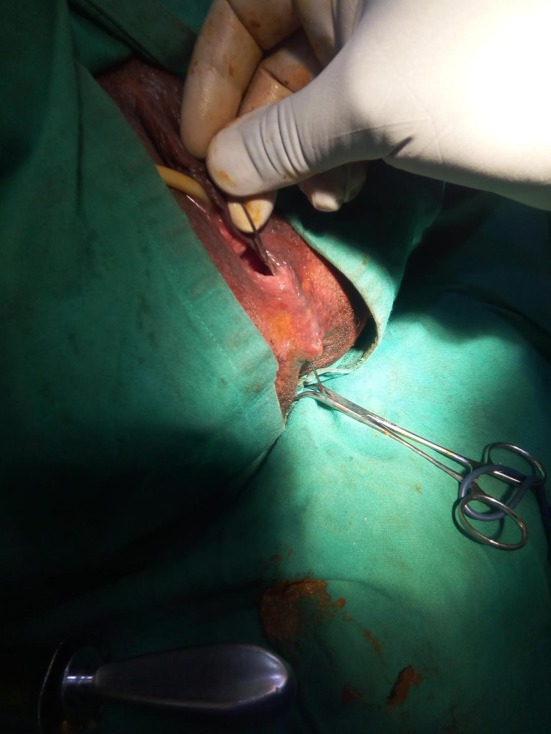
Low rectovaginal fistula delineated with probe.

**Figure 2:**
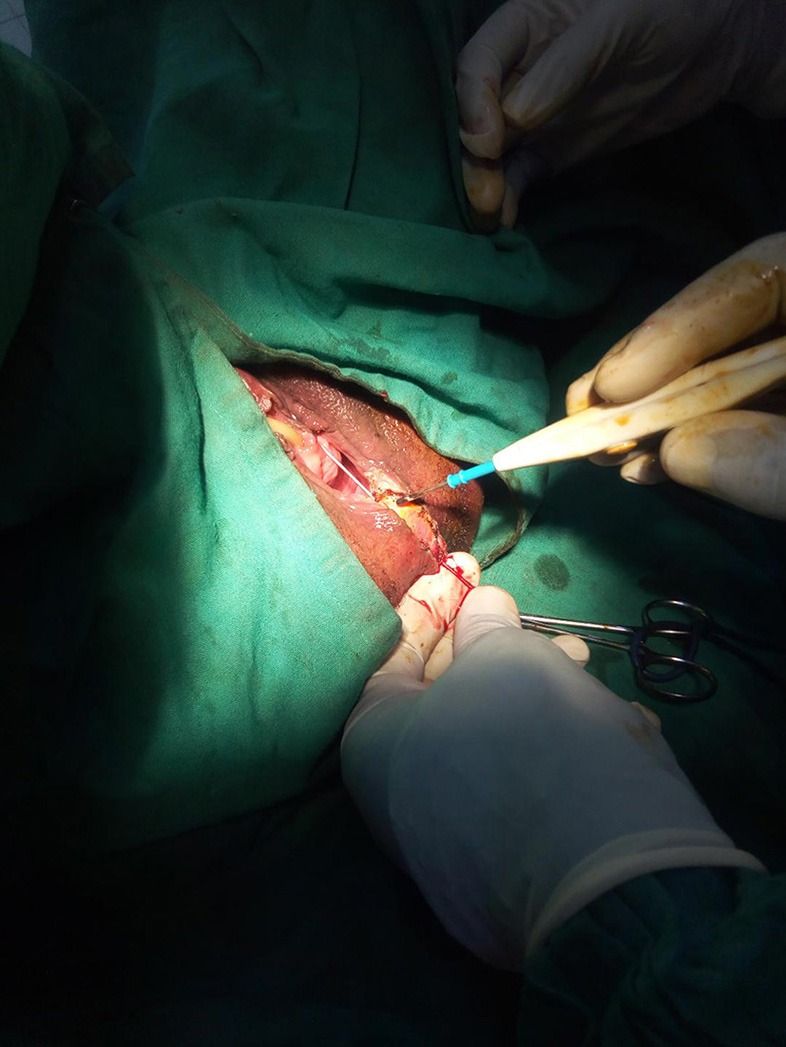
Perineal fistulotomy converting fistula to a third degree tear.

**Figure 3:**
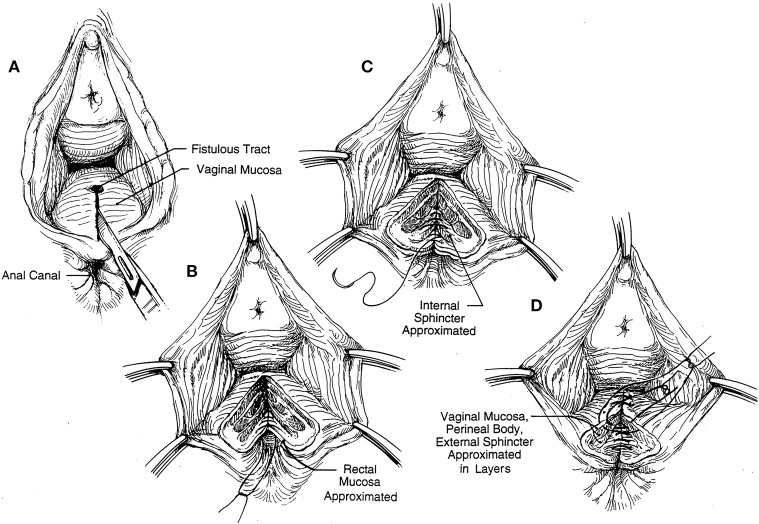
**(A–D)** Schematic representation of exposure for layered closure: the rectal mucous membrane and anal canal lining repaired followed by the rectal muscle and internal sphincter and the reunion of the retracted external anal sphincter infront (5) (with permission).

**Figure 4:**
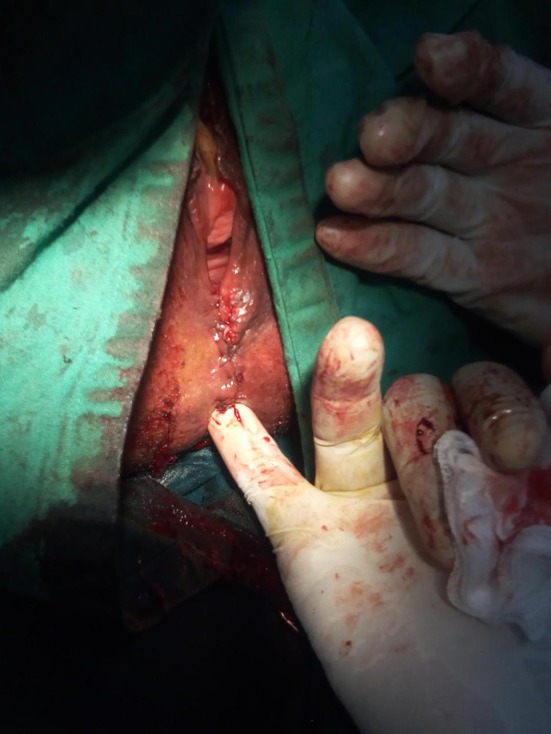
Skin over perineum closed and anal sphincter complex intact with no palpable rectovaginal fistula.

Rectal examination demonstrated a good anal tone and a pit- like defect in the anterior midline of the rectum. Proctoscopy revealed an anterior internal opening just above the dentate line which communicated with a small opening of <1 cm in diameter at 6 o'clock in the anterior edge of the vaginal fourchette and above the level of the anal sphincter. On vaginal examination, the darker mucosa in the fistula tract was apparent, and there was a whitish vaginal discharge consistent with vaginitis. A full blood count and HIV serology were normal. The diagnosis of a low rectovaginal fistula was made and she consented for repair. With the patient under spinal anesthesia, she was placed in the lithotomy position and the rectum irrigated with saline to evacuate stool. Digital rectal examination and a probe confirmed the low fistula (Figure 1), and with the Eisenhammer retractor the internal opening was easily palpable. The fistula was laid open by dividing the bridge of skin, scar tissue and muscle distal to the fistula tract, thus, converting to a third degree tear (Figure 2). Haemostasis of the posterior vaginal veins was attained with diathermy. The mucous membrane of the rectum and the lining of the anal canal were sutured with 2.0 vicryl (polyglactin) and the interrupted knots made to lie within the lumen of the bowel. The rectal muscle and internal sphincter were sutured with continuous 2.0 vicryl suture. The external anal sphincter was then reunited infront, in an overlapping manner with a horizontal mattress 2.0 vicryl suture. The vagina and the remnants of the perineal body, were reunited with an interrupted 2.0 vicryl suture inserted from within outwards with attention to symmetry (Figures 3, 4). The navicular fossa was not sutured to allow drainage of any collection whilst the skin over the perineum was closed with interrupted 2.0 nylon (Figure 4). Post-operatively, sitz baths were instituted and the patient remained in hospital for 2 days on sips of water by mouth and intravenous antibiotics. On the third day she was fed a normal diet, had a bowel movement, and discharged with 1 week of oral antibiotics, and a stool softener. At 6 months follow-up she remained well with a good anal tone, and no signs of wound infection, haematoma, or breakdown of the repair. She had a perfect Cleveland clinic continence score (CCIS) of 2/20 i.e., solids (never) 0, liquids (never) 0, flatus (sometimes) 2, use of pad (never) 0, lifestyle alteration (never) 0. Long-term follow-up was planned.

## Discussion

This was a case of a woman with a persistent simple, low rectovaginal fistula in a low resource setting. Most women with persistent symptomatic disease will not heal without surgical intervention. In addition, rectovaginal fistula from obstetric trauma is usually associated with coexistent occult sphincter injury (2, 6). In the absence of endoanal imaging and physiology there is a rationale in this setting for a fistulotomy which would convert the fistula to a third degree perineal laceration followed by layered closure (Figures 1–4). The perineal approach is not commonly used because of the inherent division of the sphincter and the unknown rate of subsequent incontinence (7). However, the sphincter muscle is the most vascular tissue between the rectum and vagina, and its use in local repair predisposes to a higher success rate and functional outcome (2, 5, 8). Not much of the anal sphincter is cut, and there is no need to explore the external anal sphincter laterally with the risk of damaging the neurovascular bundles. The technique has been evaluated in small studies and demonstrated close to a 100% success rate (5). The endorectal advancement flap is popular among colorectal surgeons. Usually the patient is placed in the prone Jack-knife position under a general anesthetic. The general principle is the excision and closure of the rectal portion of the fistula and coverage with a vascularized full-thickness rectal flap over a reapproximated rectovaginal septum on the high pressure side of the fistula (5, 9). The disadvantages of the advancement flap technique are (i) the presence of acute sepsis and a large internal opening (>2.5 cm) are contraindications for risk of anastomotic breakdown (1, 10), (ii) a heavily scarred, indurated wooden perineum would preclude adequate exposure and flap mobilization (5), (iii) it may not be durable when the anal sphincter complex is not intact (1, 2). The success rates vary widely from 29 to 100% (5, 9) which is explained by differences in technique and patient selection. No trial has been done to compare outcomes between fistulotomy and immediate reconstitution with the endorectal advancement flap procedure for low rectovaginal fistulas. A randomized trial of 55 patients with non-recurrent complex cryptoglandular fistulas comparing fistulotomy combined with immediate sphincter reconstitution and advancement flap repair had similar healing and functional outcomes (11).

## Conclusion

In the setting of no endoanal imaging and physiology, a fistulotomy and immediate reconstitution for small, low rectovaginal fistula of obstetric origin is safe and acceptable. The advantage over the advancement flap technique is that it would properly deal with an unintact anal sphincter complex which usually coexists in obstetric trauma.

## Ethics Statement

Ethical approval was waived by the University of Buea ethical committee as being a case report. Written informed consent was obtained from the patient for publication of this case report and any accompanying images. A copy of the written consent is available for review by the editor of the journal.

## Author Contributions

EW was the main author and researcher. FE carried out literature search. DE carried out literature search.

### Conflict of Interest

The authors declare that the research was conducted in the absence of any commercial or financial relationships that could be construed as a potential conflict of interest.

## Abbreviations

HIV: Human Immunodeficiency Virus
CCIS: Cleveland Clinic Incontinence Score.
